# Microglia Induce Neurotoxic IL-17+ γδ T Cells Dependent on TLR2, TLR4, and TLR9 Activation

**DOI:** 10.1371/journal.pone.0135898

**Published:** 2015-08-19

**Authors:** Katja Derkow, Christina Krüger, Paul Dembny, Seija Lehnardt

**Affiliations:** 1 Department of Neurology, Charité - Universitaetsmedizin Berlin, Berlin, Germany; 2 Cluster of Excellence NeuroCure, Charité - Universitaetsmedizin Berlin, Berlin, Germany; 3 Institute of Cell Biology and Neurobiology, Center for Anatomy, Charité - Universitaetsmedizin Berlin, Berlin, Germany; Friedrich-Alexander University Erlangen, GERMANY

## Abstract

**Background:**

Interleukin-17 (IL-17) acts as a key regulator in central nervous system (CNS) inflammation. γδ T cells are an important innate source of IL-17. Both IL-17+ γδ T cells and microglia, the major resident immune cells of the brain, are involved in various CNS disorders such as multiple sclerosis and stroke. Also, activation of Toll-like receptor (TLR) signaling pathways contributes to CNS damage. However, the mechanisms underlying the regulation and interaction of these cellular and molecular components remain unclear.

**Objective:**

In this study, we investigated the crosstalk between γδ T cells and microglia activated by TLRs in the context of neuronal damage. To this end, co-cultures of IL-17+ γδ T cells, neurons, and microglia were analyzed by immunocytochemistry, flow cytometry, ELISA and multiplex immunoassays.

**Results:**

We report here that IL-17+ γδ T cells but not naïve γδ T cells induce a dose- and time-dependent decrease of neuronal viability *in vitro*. While direct stimulation of γδ T cells with various TLR ligands did not result in up-regulation of CD69, CD25, or in IL-17 secretion, supernatants of microglia stimulated by ligands specific for TLR2, TLR4, TLR7, or TLR9 induced activation of γδ T cells through IL-1β and IL-23, as indicated by up-regulation of CD69 and CD25 and by secretion of vast amounts of IL-17. This effect was dependent on the TLR adaptor myeloid differentiation primary response gene 88 (MyD88) expressed by both γδ T cells and microglia, but did not require the expression of TLRs by γδ T cells. Similarly to cytokine-primed IL-17+ γδ T cells, IL-17+ γδ T cells induced by supernatants derived from TLR-activated microglia also caused neurotoxicity *in vitro*. While these neurotoxic effects required stimulation of TLR2, TLR4, or TLR9 in microglia, neuronal injury mediated by bone marrow-derived macrophages did not require TLR signaling. Neurotoxicity mediated by IL-17+ γδ T cells required a direct cell-cell contact between T cells and neurons.

**Conclusion:**

Taken together, these results point to a crucial role for microglia activated through TLRs in polarization of γδ T cells towards neurotoxic IL-17+ γδ T cells.

## Introduction

γδ T cells are unconventional T cells, present at approximately 1–4% in murine blood and secondary lymphoid tissue, whose T cell receptor (TCR) is composed of γ and δ chains instead of the conventional αβ TCR. These cells feature innate-like characteristics such as localization to non-lymphoid organs, activation in absence of TCR stimulation, and rapid release of inflammatory molecules upon their activation. γδ TCRs recognize ubiquitous protein and non-protein antigens without the requirement of antigen processing and being independent of the presentation of antigen via major histocompatibility (MHC) molecules [1, 2]. γδ T cells respond to conserved structures such as pathogen associated molecular patterns (PAMPs) released during infection and danger-associated molecular patterns (DAMPs) generated in the context of cell damage and stress through their TCR, Natural Killer cell receptors (NKRs), and Toll-like receptors (TLRs) [3, 4].

TLRs are pattern recognition receptors (PRRs) that recognize highly conserved PAMPs derived from viruses, bacteria, fungi, and parasites. In mice, twelve functional TLRs have been identified so far [5]. Some TLRs such as TLR1, TLR2, TLR4, TLR5, and TLR6 are localized to the cell surface and recognize mainly bacterial and fungal components, whereas TLR3, TLR7, TLR8, and TLR9 are primarily localized to endosomal membranes where they sense nucleic acids. The myeloid differentiation primary response gene 88 (MyD88) is a central adaptor molecule in TLR signal transduction. It contains the Toll-interleukin-1 (IL-1) receptor (TIR) domain, a conserved cytoplasmic region of ∼200 amino acids also shared by TLR and IL-1R family members [6]. TLRs are expressed and functional not only on antigen presenting cells, but also in other immune cells including T cells and non-immune cells including neurons [7–10]. Microglia, the resident macrophages in the brain, express and respond to all known TLRs [11]. Depending on the specific tissue analyzed including secondary lymphoid organ, skin epithelia, peritoneum, and intestinal epithelia, different subsets of murine naïve and activated γδ T cells express variable levels of various TLRs. Fang *et al*. described TLR2, TLR3, TLR4, and TLR7 mRNA expression in spleenic γδ T cells [12]. A subset (CD44highCCR6+) of peripheral murine γδ T cells has been shown to express TLR1 and TLR2—but not TLR4 [13]. Expression of TLR1, TLR2, TLR4, and TLR6 can be up-regulated in γδ T cells in response to mitochondrial DAMPs, following tissue burn injury, or by IL-23 [14–17].

γδ T cells were identified as an early source of IL-17, a proinflammatory cytokine, originally described to be produced by CD4+ Th17 T cells, in response to bacterial infection, in autoimmune diseases, and ischemia/reperfusion injury [18–22]. IL-17 production in γδ T cells can be independent of TCR activation and can be caused by activation of TLRs and cytokine receptors on γδ T cells [13, 16, 17]. Cytokines released from antigen presenting cells (APCs) activated by TLRs such as IL-1β, IL-18, and IL-23 play a crucial role in the induction of IL-17 in the absence of TCR stimulation in γδ T cells [21, 23]. TLR4-deficient γδ T cells display reduced IL-17 and IFN-γ responses during experimental autoimmune encephalomyelitis (EAE), a mouse model for multiple sclerosis [16]. Neurons can be targeted directly by CD8+ and CD4+ T cells in infection, neuroinflammatory and neurodegenerative diseases [24]. Siffrin *et al*. showed direct interaction of CD4+ Th17 cells with neurons and axons independently of TCR specificity [25].

Here, we systematically examined the neurotoxic capacity of γδ T cells and the role of microglia and TLRs in this context. γδ T cells express TLR2, TLR7, TLR9, and MyD88. However, γδ T cells do not secrete IL-17 in response to TLR ligands without microglia being present. Microglia activated through TLR2, TLR4, TLR7, or TLR9 release soluble factors including IL-1β and IL-23, inducing IL-17+ γδ T cells that display an activated phenotype. Expression of MyD88 is essential for IL-17 induction in γδ T cells by supernatants derived from microglia activated by TLRs. Both IL-17+ γδ T cells polarized by IL-1β and IL-23 *in vitro* and IL-17+ γδ T cells induced by supernatants derived from microglia activated through TLR2, TLR4, and TLR9 interact with neurons and cause cell contact-dependent neuronal cell death.

## Materials and Methods

### Animals

C57BL/6J mice were obtained from the FEM, Charité –Universitaetsmedizin, Berlin, Germany. TLR2 knock out (KO), TLR7KO, and MyD88KO mice were generously provided by Dr. S. Akira (Osaka University, Department of Host Defense, Osaka, Japan). All animals were maintained under specific pathogen-free (SPF) conditions according to the guidelines of the committee for animal care. Experimental procedures were approved by the institutional review committee Landesamt für Gesundheit und Soziales, Berlin.

### Primary culture of microglia, cortical neurons, and bone marrow-derived macrophages

Purified microglia were generated from forebrains of 0–3 day-old mice, and purified neurons were generated from mouse embryos at gestational stage 17, as described previously [26]. Murine bone marrow-derived macrophages (BMDMs) were generated as described previously using murine recombinant M-CSF (2 ng/ml) (PeproTech, Hamburg, Germany) [27].

### Isolation of γδ T cells

γδ T cells were purified from lymph nodes and spleen of 8–10 week old male C57BL/6J, TLR2KO, TLR7KO, and MyD88KO mice using the mouse TCRγ/δ^+^ T Cell Isolation Kit and magnetic cell separation (MACS) (Miltenyi Biotec GmbH, Bergisch-Gladbach, Germany). Purity of isolated γδ T cells was determined by cell surface staining of CD3 and γδ T cell receptor (γδ TCR). Purity obtained usually reached > 90% CD3+γδTCR+ cells.

### Generation of polarized IL-17+ γδ T cells

To obtain polarized IL-17+ γδ T cells, 2x10^6^/ml naïve γδ T cells were cultured for 3 days in complete RPMI (RPMI 1640 supplemented with 10% heat inactivated FCS, 1% penicillin/streptomycin, 0.05 mM β-mercaptoethanol) with IL-1β (10 ng/ml) (PeproTech, Hamburg, Germany), IL-23 (10 ng/ml) (R&D Systems), in the absence or presence of anti-CD3 (1μg/ml) and anti-CD28 (10μg/ml) (eBioscience), as described previously [21]. IL-17 production was monitored by intracellular staining of IL-17.

### *In vitro* IL-17 toxicity assay

For *in vitro* toxicity studies, indicated amounts of IL-17 (PeproTech) were added to neuronal cell cultures for indicated durations. LPS (100 ng/ml) was used as an established compound for microglia-mediated neurodegeneration, thereby testing for contamination of cell cultures with microglia. Imiquimod (10 μg/ml) or loxoribine (1mM) served as a positive control for TLR7-mediated effects. For each condition, experiments were performed in duplicates.

### Co-cultures of γδ T cells and microglia

Microglia were plated at 30x10^3^/96-well in 200 μl DMEM supplemented with 10% heat inactivated FCS, 1% penicillin/streptomycin and left to adhere overnight. After removal of 100 μl of media cells were stimulated with the TLR ligands Pam3CysSK4 (100ng/ml), imiquimod (10μg/ml) (all from InvivoGen, Toulouse, France), LPS (100ng/ml, Enzo Life Sciences GmbH, Lörrach, Germany), CpG 1668 (1μM, TIB MolBiol, Berlin, Germany) for 24 h. Subsequently, conditioned microglial supernatants were transferred to naïve γδ T cells (30x10^3^/96-well in 100 μl complete RPMI), or naïve γδ T cells were co-cultured with stimulated microglia at a 1:1 ratio. After indicated time points cells were collected for flow cytometry and supernatants were recovered for ELISA or multiplex analysis of cytokines, as indicated. TLR stimulation of bone marrow-derived macrophages was carried out likewise. For neutralization of IL-1β and IL-23, conditioned microglial supernatants were pre-incubated for 1 h at 4°C with 10 μg/ml anti-IL-1β (clone B122), anti-IL-23 (p19, clone MMp19B2) or respective isotype controls (all obtained from BioLegend, San Diego, USA) before supernatants were used for incubation of naïve γδ T cells.

### Co-cultures of γδ T cells, neurons and microglia

To generate co-cultures of neurons and polarized IL-17+ γδ T cells, half of the media was removed from DIV3-neurons (2,5x10^5^/48-well), and polarized IL-17+ γδ T cells including their culture media were added in indicated amounts and cultured for up to 96 h. Addition of complete RPMI served as a control. For co-cultures of neurons and IL-17+ γδ T cells that were induced by supernatants from microglia or BMDMs activated through TLRs, 2x10^6^/ml naïve γδ T cells were cultured for 3 days with conditioned supernatants (microglia or BMDMs stimulated for 24 h with 100 ng/ml Pam3CysSK4, 100 ng/ml LPS, 1μM CpG or no TLR ligand). Subsequently, γδ T cells and the respective supernatant were transferred to DIV3-neurons (2,5x10^5^/48-well) and cells were cultured for 5 days. For ternary co-cultures of γδ T cells, neurons and microglia, 1x10^4^ microglia were added simultaneously with γδ T cells to DIV3-neurons and cells were cultured for 3 days.

### Immunocytochemistry

Cell cultures were fixed with 4% paraformaldehyde (PFA), washed with phosphate buffered saline (PBS) and were then incubated with the primary antibody: anti-NeuN, anti-neurofilament (Merck Millipore, Darmstadt, Germany), anti-Iba1 (WAKO, Neuss, Germany) and anti-CD3 (eBioscience) overnight at 4°C. Subsequently, cell cultures were incubated with the relevant secondary antibody (all purchased from Jackson Immuno Research, West Grove, PA) for 1 h at room temperature. DAPI staining was performed, as previously described [26]. Immunofluorescence images were obtained using an Olympus BX51 microscope.

### Quantification of CNS cells *in vitro*

Viability of neurons was analyzed by quantifying NeuN+ cells in 6 to 8 fields (at 60x magnification) per cover slip, and the mean was calculated. Data are expressed as relative to control (= 100%). All quantifications were performed in a blinded manner. For analysis of apoptotic cells, DAPI+ nuclei were counted in 6 fields (at 100x magnification). After visual verification of apoptotic hallmarks such as shrinkage and fragmentation, amount of apoptotic nuclei were expressed relative to all DAPI+ nuclei in the same high power field as fold change to control.

### Cytokine Bead Assay

Multiple analyte detection of cytokines and chemokines in supernatants was performed using FlowCytomix (eBioscience).

### ELISA

Amounts of IL-17, IL-1β, IL-18, or granzyme B in cell culture supernatants were determined according to the manufacturer’s manual by ELISA (eBioscience, R&D systems).

### Flow cytometry of activation markers and intracellular cytokine staining

For cell surface staining, CD3e (clone 145-2C11), γδTCR (clone GL-3), CD25 (clone PC61.5), CD69 (clone H1.2F3) and CD62L (clone Mel-14) antibodies were obtained from eBioscience (San Diego, USA). TCR Vγ staining was performed with Vγ1.1 (clone 2.11), Vγ2 (clone UC3-10A6), Vγ3 (clone 536) obtained from BioLegend and Vγ5 (clone F2.67), kindly provided by P. Pereira (Institut Pasteur, Paris, France). The nomenclature of TCR Vγ determinants used is that of Garman et al [28]. For intracellular cytokine staining γδ T cells in co-cultures were harvested from adherent microglia before restimulation with phorbol 12-myristate 13-acetate (PMA, 10 ng/ml) and ionomycin (1 μg/ml) for 1 h and additional 3 h in the presence of brefeldin A (5 μg/ml). Cells were then washed and after fixation and permeabilisation (Cytofix/Cytoperm Kit BD Biosciences, Heidelberg, Germany) were stained intracellularly for IL-17A (clone eBio17B7) and INF-γ (clone XMG1.2) (both eBioscience). For analysis of intracellular granzyme B (clone NGZB) IC Fixation & Permeabilization Buffer was used (eBioscience). Cell surface expression of TLR1 (clone eBioTR23), TLR2 (clone 6C2) and TLR4 (clone UT41) (all from eBioscience) and intracellular expression of TLR7 (clone 66H3, Dendritics, Lyon, France), TLR9 (clone M9.D6, eBioscience) and MyD88 (clone 4d6, Imgenex, San Diego, USA) was analyzed using purified γδ T cells. Blocking of Fcγ-receptors (eBioscience) was performed before cell surface and intracellular staining. Flow cytometric analysis was performed on FACS CantoII (BD Biosciences) and analyzed with FlowJo software (TreeStar, Inc.). TLR-expression results were expressed as delta (Δ) mean fluorescent intensity (MFI) with the level of expression calculated as Δ MFI = MFI^specific antibody^-MFI^isotype^.

### Statistical analysis

Statistical differences as indicated in the figure legends were calculated using GraphPad Prism version 5.0a for Mac OS X (GraphPad Software, San Diego, USA). Differences were considered statistically significant when *p* < 0.05 with * *p* < 0.05, ** *p* < 0.01, *** *p* < 0.001.

## Results

### IL-17+ γδ T cells are neurotoxic *in vitro*

**Fig 1:**
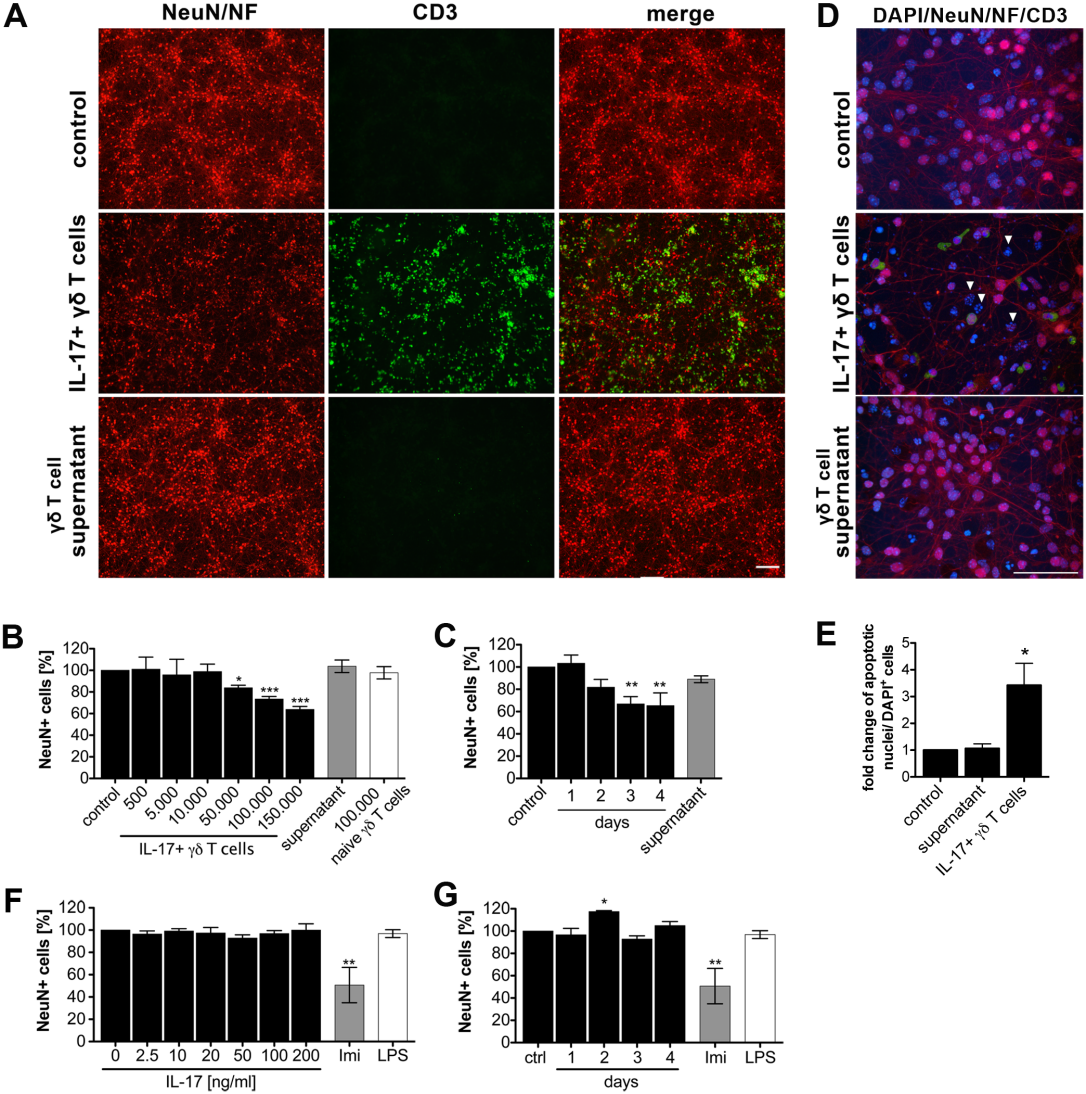
IL-17+ γδ T cells are neurotoxic *in vitro*. **(A-E)** γδ T cells were isolated from C57BL/6J mice and cultured with IL-1β (10 ng/ml), IL-23 (10 ng/ml), anti-CD3 (1μg/ml) and anti-CD28 (10μg/ml) to induce IL-17+ γδ T cells. After 3 days, polarized IL-17+ γδ T cells with their culture media or supernatants only were co-cultured with cortical neurons for 72 h. Neuronal cultures without the addition of γδ T cells served as a control. To evaluate neuronal viability cultures were subsequently immunostained with antibodies against neuronal nuclei (NeuN), neurofilament (NF) to mark neurons (both in red), CD3 to mark γδ T cells (green) and DAPI (blue). Representative images are shown for co-culture with 1x10^5^ polarized γδ T cells after 72 h, in **(A)** magnification 20x, scalebar 100μm, in **(D)** magnification 100x, scalebar 50μm, white arrowheads indicate apoptotic nuclei. In **(B, C)** γδ T cells were isolated, polarized, and co-cultured as described above at indicated concentrations for 72 h or 1x10^5^ polarized γδ T cells were co-cultured for up to 4 days. (**E)** Quantification of DAPI+ nuclei displaying apoptotic hallmarks. **(F, G)** Primary cortical neurons were incubated with recombinant IL-17 for 72 h at indicated concentrations or with 50 ng/ml for indicated time points. Neurons treated with imiquimod (10 μg/ml) or LPS (100 ng/ml) served as a positive and negative control, respectively. Cultures were then stained with NeuN Ab and DAPI. Each condition was performed in duplicate and averaged. NeuN-positive cells were quantified and expressed as relative neuronal viability. Mean ± SEM of 3–5 individual experiments, ANOVA with Dunnett´s multiple comparison post test of each time point/condition *vs*. control, (B) *p*<0.0001, (C) *p* = 0.0032, (E) *p* = 0.0151, (F) *p* = 0.7851, (G) *p* = 0.0064.

CD4+ Th17 cells were described to directly interact antigen independently with neurons and to induce axonal damage and neuronal cell death [25]. To determine whether IL-17+ γδ T cells polarized by addition of IL-1β, IL-23, α-CD3, and α-CD28 antibody can also be neurotoxic, co-cultures of IL-17+ γδ T cells and primary cortical neurons were analyzed by immunocytochemistry using markers for neurons and T cells. Assessment of the relative neuronal viability revealed that neurons co-cultured with IL-17+ γδ T cells underwent cell death (Fig 1A). This neurotoxic effect was dose- and time-dependent (Fig 1B and 1C) and required cell-cell contact, since neurons incubated with supernatants derived from IL-17+ γδ T cells were not effected by these neurotoxic effects. Furthermore, only polarized IL-17+ γδ T cells induced loss of neurons, and no reduction of neuronal numbers was detected in co-cultures of naïve γδ T cells and neurons. Increased numbers of DAPI-stained nuclei displaying hallmarks of apoptosis such as fragmentation and shrinkage (Fig 1D and 1E) in co-cultures containing IL-17+ γδ T cells but not in cultures incubated with supernatants derived from IL-17+ γδ T cells alone, confirmed neurotoxic effects of polarized IL-17+ γδ T cells. Addition of recombinant IL-17 alone to neurons did not induce loss of neurons during the observed time period, as indicated (Fig 1F and 1G).

In summary, activated IL-17+ γδ T cells interact with neurons leading to neuronal cell death *in vitro*.

### Naïve γδ T cells express TLRs but do not secrete IL-17 in response to TLR stimulation

**Fig 2:**
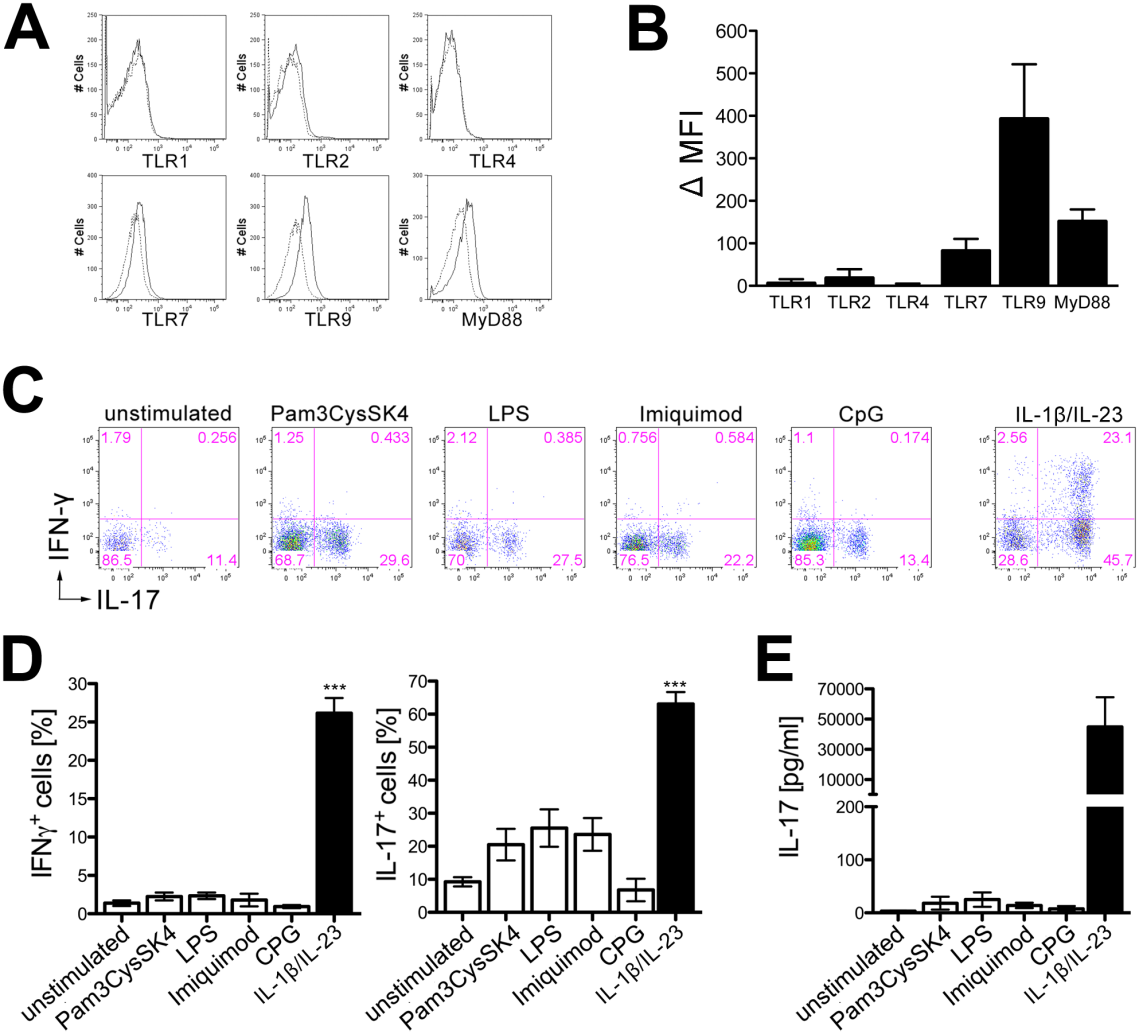
Naïve γδ T cells express TLRs but do not secrete IL-17 in response to TLR stimulation. **(A, B)** γδ T cells were isolated from C57BL/6J mice and stained at their cell surface (TLR1, TLR2, TLR4) or intracellularly (TLR7, TLR9, MyD88) with antibodies directed against the indicated TLR (solid line) and the respective isotype control (dotted line). Data are displayed as delta (Δ) mean fluorescence intensity (MFI) of the specific antibody in relation to the isotype control ± SEM from 3 to 5 individual experiments. **(C, D)** γδ T cells were isolated from C57BL/6J mice and stimulated with the TLR ligands Pam3CysSK4 (100 ng/ml), LPS (100 ng/ml), imiquimod (10 μg/ml), CpG (1 μM), or IL-1β and IL-23 (10 ng/ml each). Unstimulated cells served as a negative control. After 3 days, cells were re-stimulated and analyzed by flow cytometry for intracellular IFN-γ and IL-17 expression. Results in **(D)** are shown as mean ± SEM of 3 experiments, ANOVA with Dunnett´s multiple comparison post test of each ligand *vs*. unstimulated control. **(E)** γδ T cells were stimulated as described in **(C)**, but supernatants were collected directly after 3 days and analyzed by IL-17 ELISA. Results are shown as mean ± SEM of 3 individual experiments, ANOVA with Dunnett´s multiple comparison post test of each ligand *vs*. unstimulated control, (D) *p* = 0.3262, *p* = 0.0355, (E) *p* = 0.4647.

To examine protein expression of TLR1, TLR2, TLR4, TLR7, TLR9, and MyD88 on naïve γδ T cells flow cytometry was performed using freshly isolated and purified γδ T cells. Whereas pronounced intracellular expression of TLR7, TLR9, and MyD88 was detected, cell surface staining revealed low expression of TLR2 and almost no expression of TLR1 or TLR4 (Fig 2A and 2B). To determine if direct activation of TLRs on γδ T cells leads to IL-17 or IFN-γ production, purified γδ T cells were exposed to the TLR ligands Pam3CysSK4 (TLR2), LPS (TLR4), imiquimod (TLR7), or CpG (TLR9). γδ T cells incubated with recombinant IL-1β and IL-23 served as a positive control. Subsequent re-stimulation and flow cytometric analysis revealed that upon TLR stimulation γδ T cells did not produce IFN-γ. However, a subpopulation of γδ T cells gained the ability to produce, but not secrete, low amounts of intracellular IL-17 (Fig 2C and 2D), as determined by ELISA (Fig 2E).

Taken together, γδ T cells express TLR2, TLR7, TLR9, and the central TLR adapter molecule MyD88.

### Supernatants derived from microglia stimulated through TLRs activate naïve γδ T cells and induce expression of IL-17, but not of IFN-γ

**Fig 3:**
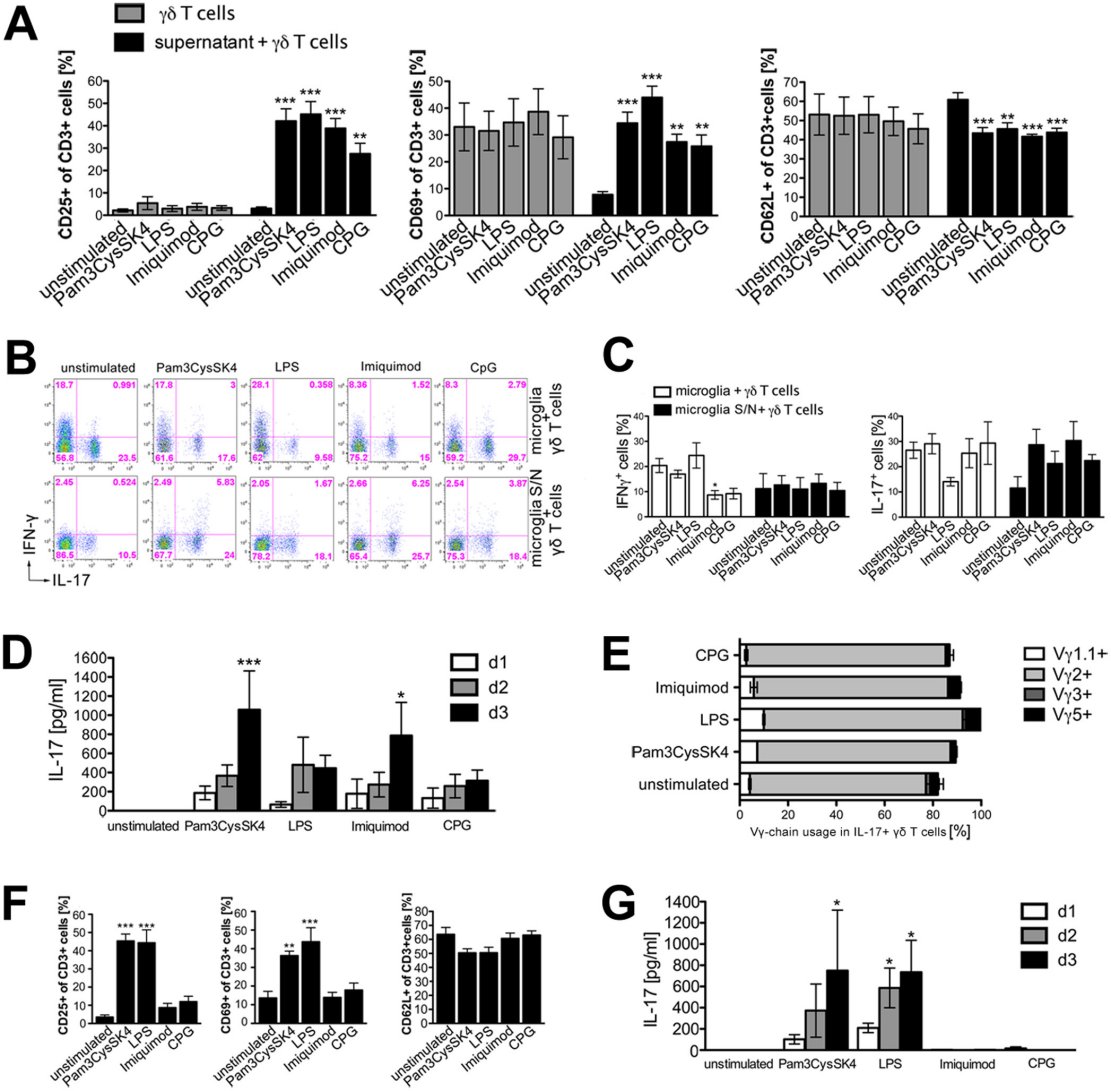
Supernatants derived from microglia stimulated through TLRs activate naïve γδ T cells and induce expression of IL-17, but not IFN-γ. **(A)** Microglia were stimulated with the TLR ligands Pam3CysSK4 (100 ng/ml), LPS (100 ng/ml), imiquimod (10 μg/ml), or CpG (1 μM) for 24 h. Microglia-conditioned supernatants were transferred to freshly isolated naïve γδ T cells, or γδ T cells were directly stimulated with the TLR ligands. Unstimulated cells served as a control. After 2 days, γδ T cells were collected and analyzed by flow cytometry regarding CD3, CD25, CD69, and CD62L expression. Each condition was performed in duplicate and averaged. Mean ± SEM of 3 to 9 individual experiments. **(B, C)** Microglia were stimulated for 24 h with TLR ligands as described in **(A)** for 24 h. Subsequently, either the microglia-conditioned supernatants were transferred to freshly isolated naïve γδ T cells or γδ T cells were co-cultured with both microglia and their supernatant. After 3 days, γδ T cells were harvested, restimulated with PMA/ionomycin, and analyzed by flow cytometry for intracellular IFN-γ and IL-17 expression. **(C)** Each condition was performed in duplicates and averaged. Mean ± SEM of 4 individual experiments. **(D)** γδ T cells were cultured with microglia-conditioned supernatant, as described in **(A).** After indicated time points supernatants were analyzed by ELISA regarding IL-17 production. Each condition was performed in duplicates and averaged. Mean ± SEM of 3 to 7 experiments. **(E)** Overview of Vγ-chain usage (Vγ1.1, Vγ2, Vγ3 and Vγ5) found on IL-17+ γδ T cells activated by supernatants derived from TLR-stimulated microglia. Mean ± SEM of 3 individual experiments. **(F)** Bone marrow-derived macrophages (BMDMs) were stimulated for 24 h with various TLR ligands as named in **(A)**. BMDM-conditioned supernatants were transferred to freshly isolated naïve γδ T cells. γδ T cells were collected after two days and analyzed by flow cytometry regarding CD3, CD25, CD69, and CD62L expression, and supernatants were collected after one, 2 and 3 days, and analyzed regarding the presence of IL-17 by ELISA **(G)**. Each condition was performed in duplicate and averaged. Mean ± SEM of 4 to 5 individual experiments. **(A)**, **(C)** and **(F)** ANOVA with Dunnett´s multiple comparison post test of each ligand *vs*. unstimulated control, (A) *p* = 0.7198, *p*<0.0001, *p* = 0.9415, *p*<0.0001, *p* = 0.9707, *p* = 0.0001, (C) *p* = 0.0061, *p* = 0.9883, *p* = 0.2590, *p* = 0.1599, (E) *p*<0.0001, *p* = 0.0004, *p* = 0.0521. **(D)** and **(G)** 2-way ANOVA with Bonferroni post test compared to unstimulated control; p*<0.05, p***<0.001.

**Fig 4:**
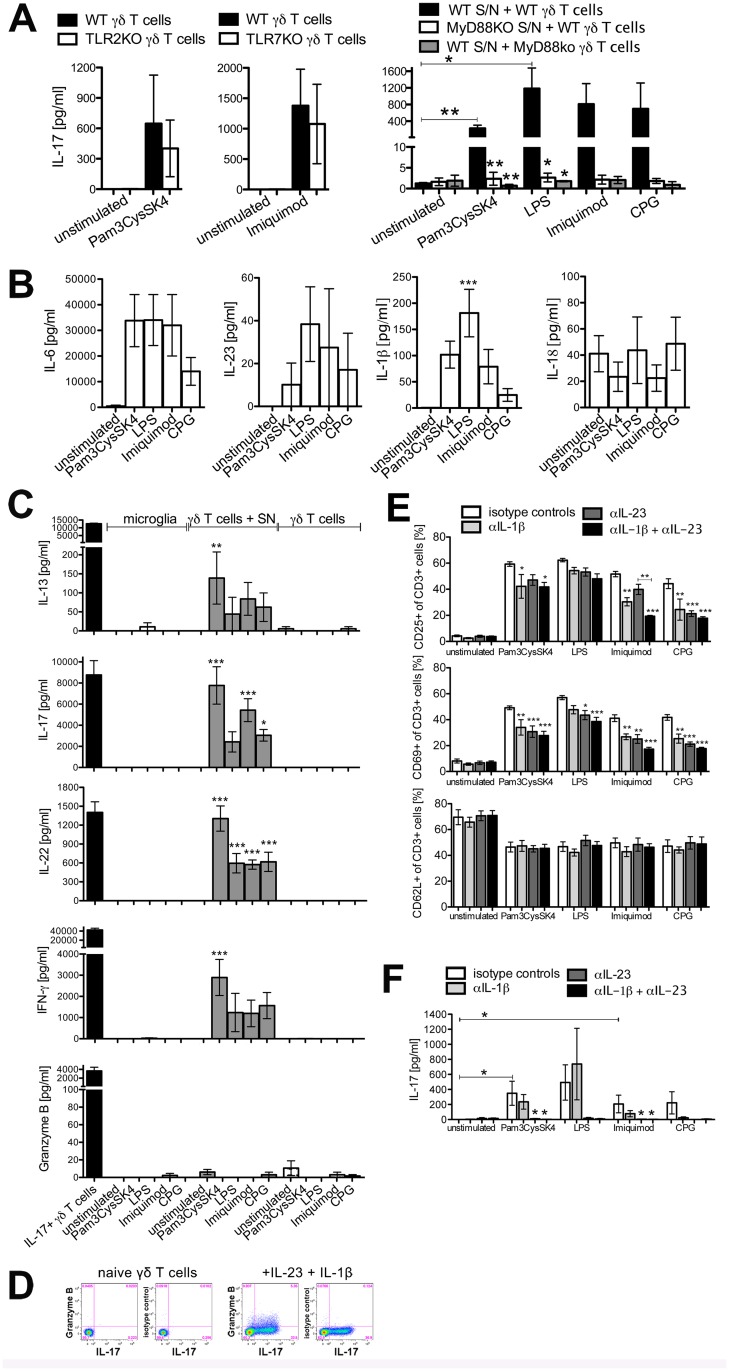
Supernatants from microglia stimulated via TLRs induce activation patterns alike to IL-17+ γδ T cells dependent on MyD88 expressed in microglia and in γδ T cells. **(A)** Wild-type (WT) or MyD88KO microglia were stimulated for 24 h with the TLR ligands Pam3CysSK4 (100 ng/ml), LPS (100 ng/ml), imiquimod (10 μg/ml) or CpG (1 μM). Microglia-conditioned supernatants were transferred to freshly isolated naïve WT, TLR2KO, TLR7KO, or MyD88KO γδ T cells. After 3 days, supernatants were collected and analyzed regarding IL-17 by ELISA. Mean ± SEM of 3–4 individual experiments. **(B, C)** WT microglia were stimulated as in **(A)** and microglia-conditioned supernatants were either used for cytokine analysis or transferred to freshly isolated naïve γδ T cells. Also, naïve γδ T cells were directly stimulated with TLR ligands as indicated or IL-1β, IL-23, anti-CD3, and anti-CD28. After 3 days, supernatants were analyzed by bead based multiplex assay or ELISA for indicated cytokines. Mean ± SEM of 3 individual experiments. Amounts of IL-1β and IL-18 were analyzed by ELISA for n = 6 individual experiments. **(D)** Naïve γδ T cells were directly analyzed. *In vitro* polarized IL-17+ γδ T cells were harvested after 3 days in culture with IL-1β, IL-23, anti-CD3, and anti-CD28, re-stimulated with PMA/ionomycin, and analyzed by flow cytometry for intracellular granzyme B expression. Representative FACS plots of n = 3 individual experiments are shown. **(E, F)** WT microglia were stimulated as in **(A)** and microglia-conditioned supernatants were preincubated with 10 μg/ml anti-IL-1β, anti-IL-23 or respective isotype controls before transfer to naïve γδ T cells. After 2 days, γδ T cells were collected and analyzed by flow cytometry regarding CD3, CD25, CD69, and CD62L expression **(E)**. After 3 days supernatants were analyzed by ELISA regarding IL-17 secretion **(F)**. Each condition was performed in duplicate and averaged. Mean ± SEM of 4 individual experiments. **(A, B, C, E, F)** ANOVA followed by Bonferroni multiple comparison post test, (A) TLR2KO *p* = 0.3340, TLR7KO *p* = 0.0989, (B) IL-6 *p* = 0.0705, IL-23 *p* = 0.5709, IL-1β *p* = 0.0011, IL-18 *p* = 0.7380, (C) IL-13 i = 0.0148, IL-17 *p*<0.0001, IL-22 *p*<0.0001, IFN-γ *p* = 0.0001, granzyme B *p* = 0.4176, (E) *p*<0.0001.

Microglia constitutively express mRNA of most of the TLRs identified so far *in vitro* [11]. Upon TLR stimulation microglia secrete the cytokines IL-1β, IL-18, and IL-23, which are known to polarize γδ T cells towards IL-17 production [11, 21, 23, 29, 30]. To investigate if microglia activated through TLRs contribute to activation and induction of IL-17+ γδ T cells, microglia isolated from C57BL/6J mice were incubated with specific ligands for TLR2 (Pam3CysSK4), TLR4 (LPS), TLR7 (imiquimod), or TLR9 (CpG) for 24 h. Subsequently, supernatants of microglial cultures were collected and transferred to freshly isolated naïve γδ T cells. After a further 48 h, cell surfaces were stained for activation markers such as CD25, CD69, and CD62L. γδ T cells incubated with the TLR ligands named above served as a control. Analysis by flow cytometry revealed that supernatants from microglia stimulated via TLRs activated γδ T cells (Fig 3A). Whereas supernatants from unstimulated microglia did not lead to up-regulation of CD25 or CD69, a significant increase of CD25+ and CD69+ γδ T cells after incubation with supernatants from microglia activated by TLR2, TLR4, TLR7, and TLR9 was detected. CD62L, a marker for naïve T cells, was significantly down-regulated upon incubation of γδ T cells with supernatants from TLR-stimulated microglia. This activation pattern observed was not due to direct activation of γδ T cells by TLR agonists remaining in the cell culture media, since changes in regulation of CD25, CD69, and CD62L, respectively, after TLR stimulation were not observed in control cultures of γδ T cells that were directly activated by TLR ligands. Next, we investigated whether co-cultures of microglia stimulated through TLRs with γδ T cells or supernatants of TLR-stimulated microglia also induce IFN-γ or IL-17 in γδ T cells (Fig 3B and 3C). IFN-γ production was neither induced in γδ T cells in the presence of microglia nor by incubation with supernatants derived from microglia activated through TLR2, TLR4, TLR7, or TLR9. However, IFN-γ production was rather down-regulated in co-cultures of γδ T cells and microglia after TLR7 stimulation. The presence of unstimulated microglia alone enabled γδ T cells to produce IL-17, and this effect was not further increased by precedent TLR stimulation. On the contrary, stimulation of microglia by LPS preceding the co-culturing with γδ T cells led to a decreased production of IL-17, although these results did not reach statistical significance in our experimental set up. Only if γδ T cells were incubated with supernatants from microglia activated through TLRs, intracellular production of IL-17 in γδ T cells was increased. Since IL-17 has to be secreted to exert its biological functions, we next determined whether IL-17 was not only produced, but also secreted by γδ T cells (Fig 3D). To this end, supernatants were collected after one, two, or three days of culturing and subjected to IL-17 ELISA. Supernatants from microglia stimulated through TLR2 or TLR7 induced a time-dependent secretion of IL-17 in γδ T cells. To assess the γδ T cell subtype classified on the base of Vγ-chain usage being responsible for IL-17 production [13], we investigated the expression of Vγ1.1, Vγ2, Vγ3 and Vγ5 on IL-17+ γδ T cells incubated with supernatants from TLR-activated microglia by flow cytometry. γδ T cells utilizing the TCR chain Vγ2 were identified as the main producers of IL-17 (Fig 4E).

In summary, microglia activated via TLR2, TLR4, TLR7, and TLR9 secrete soluble factors inducing IL-17+ γδ T cells that exhibit an activated phenotype.

### Compared to microglia supernatants from BMDMs stimulated via TLRs induce different patterns of activation in γδ T cells

To examine if induction of IL-17 in γδ T cells is specific for microglia, or can also be achieved by peripheral macrophages, bone marrow-derived macrophages (BMDMs) were incubated with the TLR ligands Pam3CysSK4, LPS, imiquimod, and CpG *in vitro*. Subsequently, supernatants were collected after 24 h and were used for the incubation of γδ T cells. After 48 h, γδ T cells were analyzed in terms of activation using the markers CD25, CD69, CD62L, and were tested for amounts of IL-17 in the supernatants. In contrast to supernatants derived from microglia activated through TLRs, only supernatants from BMDMs activated by Pam3CysSK4 and LPS, but not by imiquimod or CpG, induced significant up-regulation of CD25 and CD69 (Fig 3F). Likewise, IL-17 secretion was only observed after incubation with supernatants derived from BMDMs activated through TLR2 and TLR4, but not TLR7 or TLR9 ligands (Fig 3G). Although it is known that BMDMs can respond to TLR7 and TLR9 [31], stimulation with supernatants of BMDMs activated by imiquimod or CpG did not result in the induction of IL-17+ γδ T cells.

These results indicate that activation of γδ T cells through peripheral immune cells differs from activation of γδ T cells through microglia, the major immune cells in the CNS.

### Supernatants from microglia stimulated via TLRs induce activation patterns alike to IL17+ γδ T cells dependent on MyD88 expressed in microglia and γδ T cells

As outlined above, mere stimulation of γδ T cells through TLRs did not induce IL-17 secretion. Yet, stimulation of microglia through TLRs drove the production of soluble components secreted from microglia, which then induced IL-17 secretion in γδ T cells. This effect may be due to stimulation of TLRs on γδ T cells by TLR ligands still present in the microglial supernatant and to effector molecules secreted by microglia activated through TLRs. Therefore, wild-type microglia were stimulated with Pam3CysSK4 and imiquimod that elicited a strong IL-17 response in γδ T cells (see Fig 3D). After 24 h, supernatants were collected and TLR2KO and TLR7KO γδ T cells were incubated with these supernatants. Under these circumstances no reduction of IL-17 induction was detected (Fig 4A). Similar observations were made using γδ T cells derived from TLR4KO mice (data not shown). To verify the requirement of TLR ligand-induced activation of microglia and to elucidate the role of IL-1 receptor signaling in this context, MyD88KO microglia and MyD88KO γδ T cells were analyzed after TLR2, TLR4, TLR7, or TLR9 stimulation. IL-17 production by γδ T cells was dependent on both functional MyD88 in microglia and functional MyD88 in γδ T cells, indicating that microglia require TLR signaling to secrete molecules needed for IL-17 induction in γδ T cells and suggesting that γδ T cells require functional MyD88 signaling, potentially via activation of the IL-1R in γδ T cells.

IL-1β, IL-18, and IL-23 play a key role in the induction of IL-17 in γδ T cells [21, 23]. To elucidate if any of these molecules are secreted by microglia, supernatants derived from microglia were collected at 24 h after stimulation of TLR2, TLR4, TLR7, and TLR9 with the respective agonists. Subsequently, cytokines were determined by ELISA and multiplex assay. Activation of microglia through TLR2, TLR4, TLR7, and TLR9 induced high expression of IL-6, IL-1β, and varying amounts of IL-23, but not IL-18 (Fig 4B).

To investigate which other cytokines are produced by IL-17+ γδ T cells supernatants from microglia stimulated by TLR ligands were tested by multiplex assay and ELISA. A striking similarity between polarized γδ T cells and γδ T cells induced by microglial supernatants in terms of the cytokine response including IL-13, IL-17, IL-22, and IFN-γ was observed (Fig 4C). Supernatants from microglia activated with Pam3CysSK4, LPS, imiquimod as well as CpG induced IL-13, IL-17, IL-22, and IFN-γ in γδ T cells, albeit at different levels depending on the specific activated TLR involved. None of these cytokines were induced by direct TLR stimulation of γδ T cells or of microglia, suggesting an induction in γδ T cells by molecules secreted from microglia after TLR stimulation. Supernatants of microglia activated through TLRs did not induce the production of granzyme B, a cytolytic effector molecule mediating neuronal injury by CD4+ Th17 cells [32], in γδ T cells, as was observed if γδ T cells were incubated with IL-β and IL-23 (Fig 4C and 4D).

To determine whether IL-1β and IL-23 derived from microglia contribute to the activation of γδ T cells, microglial supernatants were treated with neutralizing antibodies to IL-1β, IL-23, both cytokines, or isotype control antibodies before addition to γδ T cells. Whereas no effect on the expression of CD62L was detected, inhibition of IL-1β or IL-23 led to a reduction of CD25 and CD69 expression on γδ T cells. Simultaneous inhibition of IL-1β and IL-23 had no additive effect and a reduction to baseline levels of γδ T cells incubated with supernatant derived from unstimulated microglia was not achieved (Fig 4E). A significant reduction of IL-17 secretion was achieved by blocking IL-23 alone and together with IL-1β in supernatants derived from microglia activated through TLR2 or TLR7 (Fig 4F).

In summary, these results indicate a role for IL-1β and IL-23, but not IL-18, secreted from microglia in the activation of γδ T cells and induction of IL-17 production.

### Supernatants from microglia stimulated via TLR2, TLR4, or TLR9 induce neurotoxic γδ T cells

**Fig 5:**
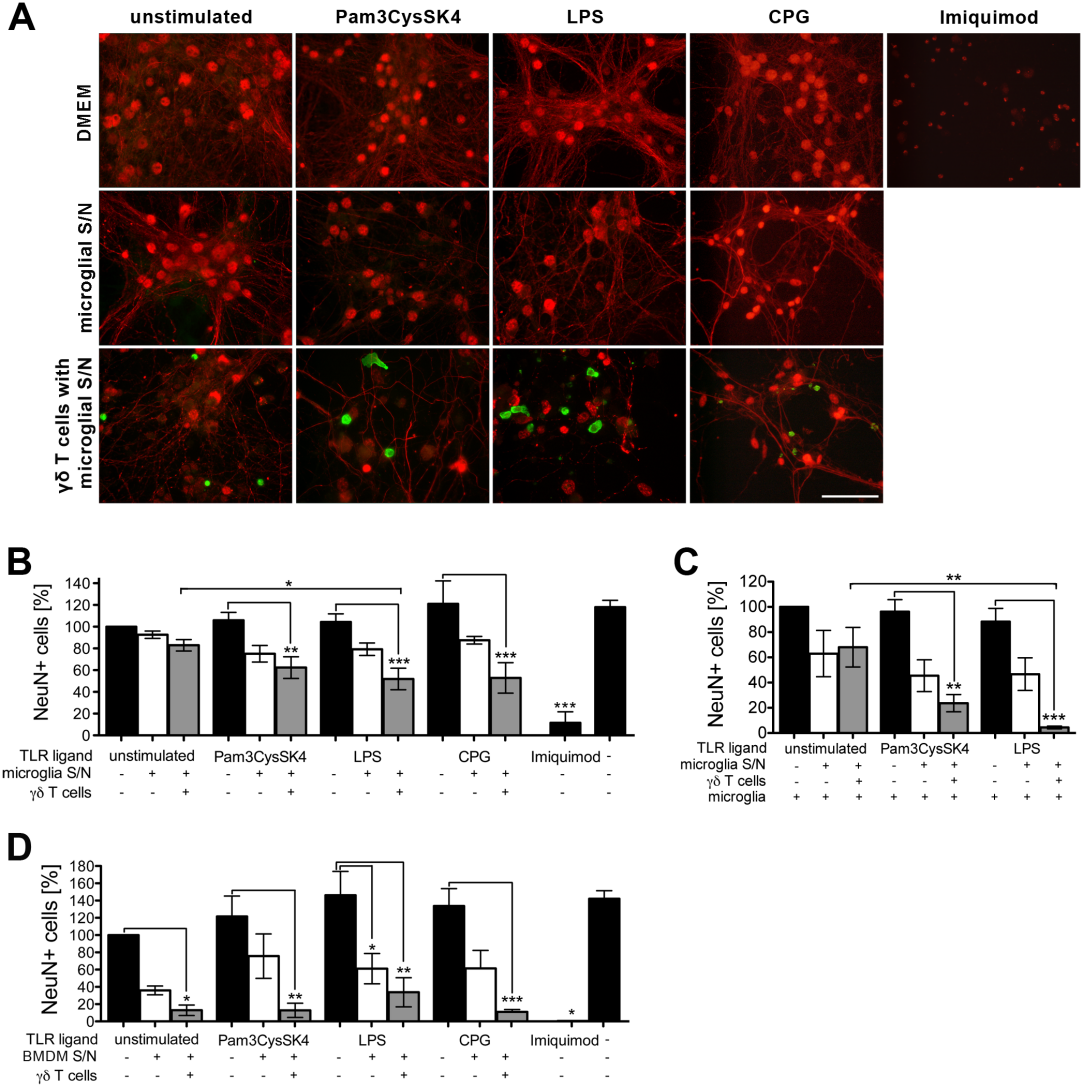
Supernatants from microglia stimulated via TLR2, TLR4, or TLR9 induce neurotoxic γδ T cells. **(A)** Microglia were stimulated with Pam3CysSK4 (100 ng/ml) LPS (100 ng/ml) or CpG (1 μM) for 24 h. Unstimulated cells served as a control. Microglia-conditioned supernatants were transferred to freshly isolated naïve γδ T cells. After 3 days, γδ T cells or microglia-conditioned supernatant only were supplemented with cortical neurons for additional 5 days. Neuronal cultures without γδ T cells in the presence of Pam3CysSK4 (100 ng/ml), LPS (100 ng/ml), CpG (1 μM) or imiquimod (10 μg/ml) alone, served as controls. Subsequently, cultures were immunostained with antibodies against CD3 (γδ T cells, green), NeuN, and neurofilament (neurons, red), magnification 100x, scale bar 50 μm. In **(C)** 1*10^5^ microglia were added to neuronal cultures concurrent with γδ T cells. **(D)** BMDMs were stimulated with Pam3CysSK4 (100 ng/ml), LPS (100 ng/ml) or CpG (1 μM) for 24 h. Unstimulated cells served as a control. BMDM-conditioned supernatants were transferred to freshly isolated naïve γδ T cells. After 3 days, γδ T cells or microglia-conditioned supernatant only were supplemented with cortical neurons for additional 5 days. Neuronal cultures without γδ T cells in the presence of Pam3CysSK4 (100 ng/ml), LPS (100 ng/ml), CpG (1 μM) or imiquimod (10 μg/ml) alone served as controls. Subsequently, cultures were immunostained as in **(A)**. **(B, C, D)** NeuN-positive cells were quantified and expressed as relative neuronal viability. Each condition was performed in duplicate and averaged. Mean ± SEM of 3–5 individual experiments with ANOVA followed by Bonferroni multiple comparison post test, (B) *p*<0.0001, (C) *p*<0.0001, (D) *p*<0.0001.

To elucidate if IL-17+ γδ T cells induced by supernatants from microglia activated through TLRs can be neurotoxic, γδ T cells were incubated with supernatants of cultured microglia stimulated with Pam3CysSK4 (TLR2), LPS (TLR4), or CpG (TLR9) for 3 days. Supernatants from unstimulated microglia served as a control. Subsequently, γδ T cells were co-cultured with cortical neurons, and neuronal viability was evaluated after another 5 days. As a further negative control, neurons were incubated with the respective TLR ligand or with microglial supernatants only. The TLR7-specific ligand imiquimod, which is known to induce major cell-autonomous neurotoxic effects [8, 33], was used as a positive control for neurotoxicity (Fig 5A and 5B). Neuronal viability was decreased when neurons were cultured with supernatants derived from microglia stimulated by Pam3CysSK4, LPS, or CpG. This neurotoxic effect was significantly enhanced when γδ T cells were added. In particular, a distinct reduction of neuronal numbers in the presence of γδ T cells was detected when microglia had been stimulated via TLR4 beforehand (Fig 5B). We also evaluated whether γδ T cell neurotoxicity is effective in neurons lacking TLR7 (TLR7KO) (S1A Fig). As observed in cultures of wild-type neurons, relative neuronal viability was significantly decreased when TLR7KO neurons were cultured with γδ T cells stimulated by supernatants from microglia activated through TLR2 or TLR4. Since imiquimod is known to interfere with adenosin receptor signaling pathways [34], eventually leading to cell death of TLR7KO neurons independently of TLR7 in our experimental set-up, loxoribine, also an established TLR7 ligand, but not interfering with other pathways [8, 35], was used additionally to confirm TLR7KO specificity (S1B Fig).

To determine whether the presence of microglia has an impact on to the neurotoxic capacity of activated γδ T cells, the experimental set-up with wild-type neurons as described above was repeated, and co-cultures of γδ T cells and neurons were additionally supplemented with microglia (Fig 5C). The presence of microglia in cultures containing γδ T cells activated by supernatants from microglia stimulated through TLR4 significantly decreased relative neuronal viability as soon as after 3 days of incubation to 4.45 ± 1.06% (mean ± SEM), whereas 5 days of incubation were required to decrease neuronal viability to 51.88 ± 9.92% (mean ± SEM) when co-cultures of neurons and γδ T cells activated by supernatants from TLR4-stimulated microglia alone were investigated (see Fig 5B).

To investigate whether supernatants from BMDMs activated through TLR2, TLR4, or TLR9, which differed from microglial supernatants in terms of activating γδ T cells (see Fig 3F and 3G), also differed in induction of γδ T cells with neurotoxic features, γδ T cells were incubated with supernatants from BMDMs stimulated with Pam3CysSK4 (TLR2), LPS (TLR4), or CpG (TLR9) for 3 days. Supernatants from unstimulated BMDMs served as a control. Subsequently, γδ T cells were co-cultured with cortical neurons, and neuronal viability was evaluated after 5 days. As a further control, neurons were incubated with the respective TLR ligand or with BMDM supernatants only (Fig 5D). Both γδ T cells activated with unstimulated BMDM supernatant and γδ T cells activated with supernatants from BMDMs stimulated through TLR2, TLR4, or TLR9 lead to a decrease in neuronal viability, indicating that TLR activation was not required for γδ T cell-mediated neurotoxicity in this context.

Taken together, microglia activated through TLR2, TLR4, or TLR9 can contribute to activation of γδ T cells and promote their maturation into neurotoxic effector cells *in vitro*.

## Discussion

The current study demonstrates that supernatants from microglia activated through TLRs induce neurotoxic IL-17+ γδ T cells. It is well established that IL-17 induces the expression of pro-inflammatory genes in fibroblasts, endothelial cells and macrophages, permeabilizes the blood brain barrier and mediates recruitment of neutrophils into the CNS [32, 36, 37]. Originally, IL-17 was described to be produced by CD4+ Th17 cells, but meanwhile has also been detected to be secreted by CD8+ T cells, innate lymphoid cells, natural killer cells, and γδ T cells. Moreover, γδ T cells have been identified as major source of IL-17 in response to bacterial infection, in autoimmune diseases, and in ischemia/reperfusion injury [18–22]. In EAE, a mouse model for multiple sclerosis, IL-17+ γδ T cells play a pathogenic role in the initiation of the disease. Both γδ T cell-depleted and TCR^-/-^ mice exhibit a reduced disease severity and delayed onset of EAE. Further, IL-17 derived from γδ T cells helps to promote IL-17 production in CD4+ T cells [21, 38]. Accordingly, in humans, clonally expanded γδ T cells were detected in acute MS plaques and chronically demyelinated areas [39, 40]. Also, the frequency of CD161highCCR6+ γδ T cells in the cerebrospinal fluid of patients with clinically isolated syndrome/MS in relapse is increased, and these cells are capable of producing IL-17 [41]. However, our *in vitro* studies reveal that IL-17 is not neurotoxic by itself, but toxic effects require activated IL-17+ γδ T cells. In line with our results Wang *et al*. showed that addition of IL-17 to cultured hippocampal neurons under normoxic conditions had no effect on neuronal cell death, whereas oxygen-glucose depriviation stress led to up-regulation of IL-17 receptor (IL-17R) on neurons and neuronal apoptosis after addition of IL-17 [42]. Similarly, supernatant from peripheral blood mononuclear cells stimulated with PMA/ionomycin, which contains not only IL-6 and IFN-γ but also high levels of IL-17, causes apoptosis of cerebellar granule neurons. However, the presence of astrocytes producing IL-17 exerts neuroprotection [43]. On the contrary, studies by Kang *et al*. showed that neuronal NF-κB activator 1 (Act1), an intracellular adaptor in IL-17 signaling, is dispensable for Th17-mediated EAE pathogenesis [44]. Thus, IL-17 is a characteristic cytokine expressed in γδ T cells modulating neuroinflammation, but is not the causative factor of neurotoxicity [45].

We have detected TLR2, TLR7, TLR9, and MyD88, but hardly any TLR1 or TLR4 on γδ T cells isolated from peripheral lymph nodes and spleen. However, naïve peripheral CD44+ γδ T cells were found to express TLR2, but not TLR4 on protein level [13]. Further, CD44highCCR6+ γδ T cells express TLR1 and TLR2 mRNA but not TLR4 mRNA. It was recently shown that TLR1, TLR2, TLR4, TLR6, and TLR9 are expressed in IL-17+ γδ T cells and that TLR1, TLR2, TLR4, and TLR6 are up-regulated after stimulation with IL-23 [16, 17]. Murine γδ T cells directly respond to TLR2 and TLR4 by proliferating and secreting cytokines, independently of TCR activation [13, 16, 17]. In general, IL-17 production by γδ T cells after sole TLR stimulation is low, but can be enhanced by the addition of IL-23 [13, 16, 17, 46]. In accordance with these findings, we observed production of intracellular IL-17, but detected only low levels of IL-17 secreted by γδ T cells after TLR stimulation. However, since the purity of γδ T cell preparation in our experiments usually was >90%, it seems unlikely that contaminating APCs respond to TLR stimulation with IL-23 or IL-1β production to induce IL-17 in γδ T cells.

γδ T cells express IL-1R1 and IL-23R, and IL-1β can synergize with IL-23 to promote IL-17, -22, and IFN-γ release from γδ T cells [20, 21, 23, 47]. Therefore, it is conceivable that activation of microglia through TLRs and the subsequent release of IL-23 and IL-1β play a role in generating IL-17+ γδ T cells. In accordance with this, activation of microglia through TLR2, TLR4, TLR7, and TLR9 induced the expression of IL-1β, and IL-23 in our study. Likewise, IL-6, important for differentiation of CD4+ Th17 cells, was detected at high levels, but has previously been shown to be dispensable for IL-17 induction in murine γδ T cells [13]. In contrast, IL-18, which together with IL-23 induces IL-17 in γδ T cells and which is released by microglia in response to infection was not detected in TLR-activated microglial supernatants [23, 48, 49]. The fact that IL-1β and IL-23 as well as supernatants derived from microglia activated through TLRs induce a highly similar cytokine pattern including IL-13, IL-17, IL-22, and IFN-γ points to a role of IL-1β and IL-23 secreted from microglia in IL-17 induction in γδ T cells. We show here that supernatants from microglia stimulated by TLR2-, TLR4-, TLR7-, and TLR9-specific ligands activate γδ T cells as determined by CD25, CD69 up-regulation and CD62L down-regulation, and induce substantial production and secretion of IL-17, but not IFN-γ in γδ T cells. This effect is due to soluble factors released from microglia since supernatants from microglia activated through TLRs were sufficient to stimulate cytokine production in γδ T cells. We here present data that IL-1β and IL-23 contribute to the activation of γδ T cells, and that IL-23 alone is sufficient to induce IL-17 secretion. Supernatants from TLR-activated microglia enhanced intracellular IL-17 production by γδ T cells to the level that was induced by co-culturing them with unstimulated microglia. However, in the presence of unstimulated microglia γδ T cells do not secrete IL-17 (data not shown). Thus, in a pathophysiological context entry of γδ T cells into the CNS may cause intracellular IL-17 production via contact with microglia, and this effect may not require TLR signaling. Yet, a TLR stimulus to activate resting microglia to produce IL-1β and IL-23 may be necessary to evoke IL-17 secretion by γδ T cells.

Surprisingly, while IL-17 production by γδ T cells was increased by incubation with supernatants derived from TLR-stimulated microglia, the presence of microglia activated through TLR4, TLR7, or TLR9 reduced the production of IL-17 and IFN-γ by γδ T cells. Since activated microglia do not only contribute to neurodegeneration, but can also play a beneficial role in neuroprotection [50], one can speculate that the direct interaction of the CNS infiltrating γδ T cells with TLR-activated microglia e.g. through cell surface molecules rather counteracts the activation of γδ T cells, thereby dampening the inflammatory immune response. An alternative pathophysiological scenario would be one in which cytokines initially produced by γδ T cells in response to soluble factors derived from TLR-stimulated microglia may promote a subsequent shift towards a neuroprotective microglial activation status. Thereby, a limitation of the inflammatory response generated by γδ T cells would be finally achieved.

We found that co-stimulation by secretory molecules and agonists specific for TLR2 and TLR7 was not necessary for IL-17 induction in γδ T cells since γδ T cells from the respective TLR-deficient mice were still able to produce IL-17 upon incubation with microglial supernatants. Still, signaling that leads to the production of IL-17 must involve MyD88, since MyD88KO γδ T cells were not capable of producing IL-17. In general, MyD88 signaling is required for both TLR and IL-1R1 signal transduction [51, 52]. Yet, MyD88-deficient CD4+ T cells were described to be impaired in Th17 differentiation and IL-1 as well as IL-23 signaling, was demonstrated to depend on MyD88 [53]. Previously, MyD88-deficient mice were shown to exhibit an impaired generation of IL-17+ γδ T cells in response to malaria infection [54]. Accordingly, the findings of our current study indicate that functional MyD88 is essential for IL-17 induction in γδ T cells by supernatants derived from TLR-activated microglia, additionally pointing to the involvement of IL-1β and IL-23 secreted from microglia.

Induction of IL-17 in γδ T cells in the presence of other immune cells such as bone marrow-derived dendritic cells has been described before [23]. We show here that microglia, the major immune cells of the brain, likewise feature similar properties. In detail, supernatants from microglia stimulated through TLR2, TLR4, TLR7, or TLR9 resulted in activation and IL-17 production of γδ T cells. This effect on γδ T cells seems to be cell type-specific since supernatants derived from equally activated bone marrow-derived macrophages (BMDMs) induced these effects only by activation of TLR2 or TLR4, but not by activation of TLR7 or TLR9. Moreover, microglia required TLRs to induce a neurotoxic γδ T cell phenotype, whereas BMDMs induced such a phenotype independently on TLR signaling. Accordingly, previous studies performed by other groups demonstrated that the sole presence of BMDMs causes neuronal dysfunction and ultimately neurodegeneration [55].

We observed neurotoxic effects of IL-17+ γδ T cells *in vitro*, and these effects required cell-cell contact between neurons and T cells. In a mouse model of ischemia/reperfusion injury infiltrating macrophages were the main source of IL-23 after ischemia, promoting IL-17 production of γδ T cells. γδ T cells were shown to localize to the infarct boundary zones [22, 36]. Localization of γδ T cells to an area where neuronal cell death is observed indicates the possibility of direct interaction of activated IL-17+ γδ T cells with neurons. A prolonged interaction of CD4+ Th17 cells with axons and cell contact-dependent localized Ca2+ increase leading to neuronal injury was reported [25]. Also, neuronal apoptosis and expression of Fas on neurons and FasL on infiltrating Th17 cells were observed in a rat model of Alzheimer´s disease [56]. However, in our experiments neither IL-17+ γδ T cells induced by addition of IL-1β and IL-23 nor those induced by supernatants from microglia stimulated through TLRs expressed FasL (data not shown). As mentioned above, neurotoxic effects mediated by IL-17+ γδ T cells required a direct contact between T cells and neurons, and neither supernatant derived from IL-17+ γδ T cells nor addition of recombinant IL-17 alone, was sufficient to decrease neuronal viability. Interestingly, a spatial proximity between IL-17+ γδ T cells and neurons was frequently observed *in vitro* (data not shown). Close contact between γδ T cells and neurons may point to the involvement of other, yet unidentified, cellular mechanisms mediating neurotoxicity. Granzyme B might be one effector molecule mediating neuronal injury as has been described for CD4+ Th17 cells [32]. However, in our studies supernatants from TLR-activated microglia did not induce production of granzyme B in γδ T cells, as did activation of γδ T cells with IL-β and IL-23. Also, CD4+ Th1, Th17, CD8+ T cells as well as NK cell lysates were reported to induce neuronal damage via the induction of microtubule axonal destabilization mediated by lytic granules [57]. Further studies are required to elucidate whether IL-17+ γδ T cells induced by TLR-activated microglia use a similar mechanism.

In summary, soluble factors, including IL-1β and IL-23 secreted from microglia stimulated by TLR-specific ligands, induce MyD88-dependent activation of γδ T cells. Such activated γδ T cells resemble IL-17+ γδ T cells generated by the addition of IL-1β and IL-23. The presence of IL-17+ γδ T cells but not naïve γδ T cells, results in toxic effects towards neurons that require a direct cell-cell contact between neurons and γδ T cells.

## Supporting Information

S1 Fig Supernatants from microglia stimulated via TLRs induce γδ T cells that are neurotoxic towards TLR7KO neurons. **(A)** Microglia were stimulated with Pam3CysSK4 (100 ng/ml) or LPS (100 ng/ml) for 24 h. Unstimulated cells served as a control. Microglia-conditioned supernatants were transferred to freshly isolated naïve γδ T cells. After 3 days, γδ T cells or microglia-conditioned supernatant only were supplemented with cortical TLR7KO neurons for additional 5 days. Neuronal cultures without γδ T cells in the presence of Pam3CysSK4 (100 ng/ml), LPS (100 ng/ml) or imiquimod (10 μg/ml) alone served as controls. Subsequently, cultures were immunostained with antibodies against CD3, NeuN, and neurofilament. NeuN-positive cells were quantified and expressed as relative neuronal viability. Each condition was performed in duplicate and averaged. Mean ± SEM of 3–5 individual experiments with ANOVA followed by Bonferroni multiple comparison post test, *p*<0.0001. **(B)** Primary cortical neurons derived from wild-type (WT) or TLR7KO mice were stimulated with loxoribine (1 mM). Untreated cells served as controls. After 5 days cultures were immunostained with antibodies against NeuN and neurofilament. NeuN-positive cells were quantified and expressed as relative neuronal viability. Each condition was performed in duplicate and averaged. Mean ± SEM of 2–3 individual experiments with ANOVA followed by Bonferroni multiple comparison post test, *p*<0.0001.
(TIF)
