# Supplementary figures and images for: Microglia Induce Neurotoxic IL-17+ γδ T Cells Dependent on TLR2, TLR4, and TLR9 Activation
Source: PLoS One. 2015 Aug 19;10(8):e0135898. doi: 10.1371/journal.pone.0135898 (PMC4545749; doi:10.1371/journal.pone.0135898)

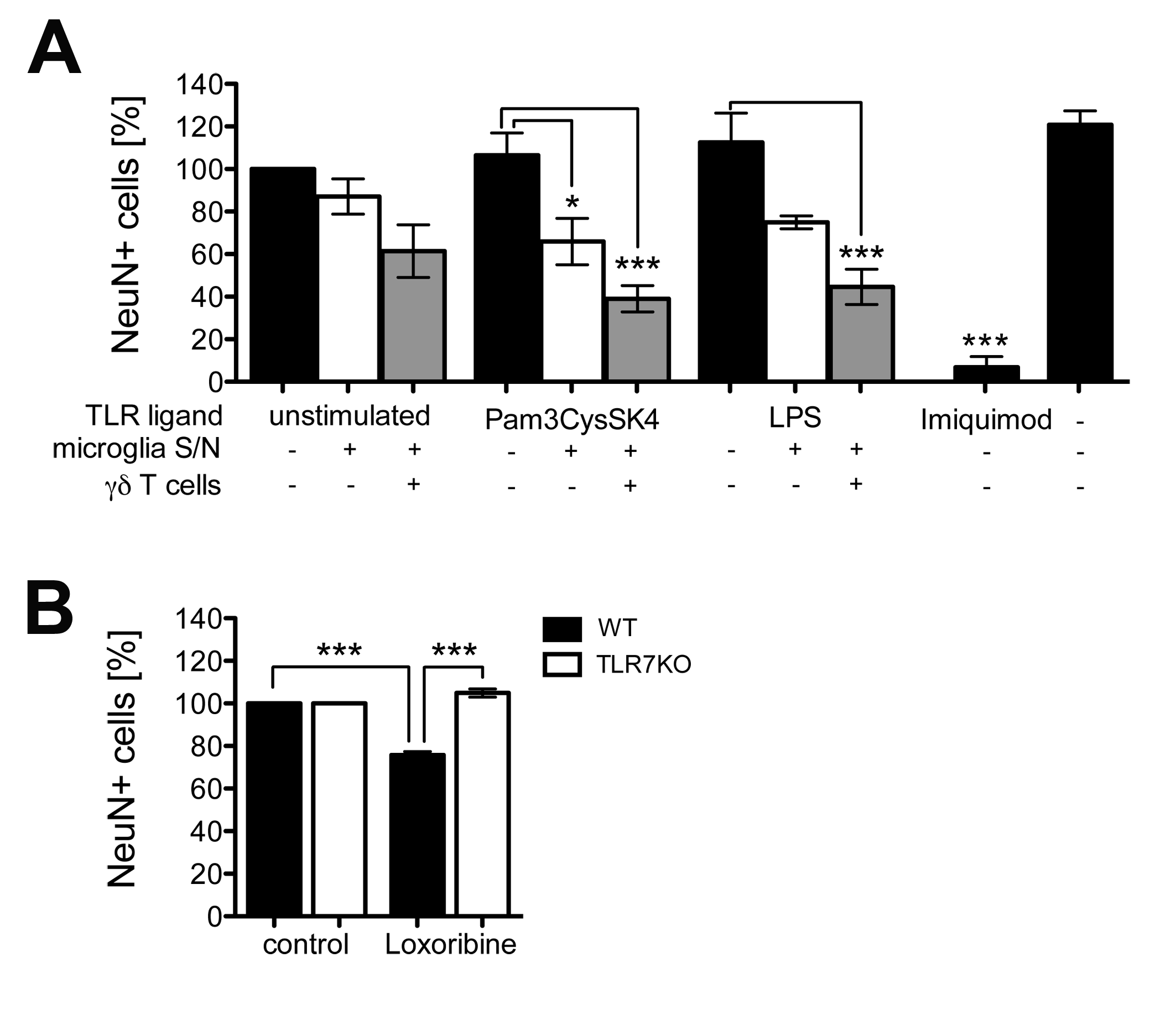

Supplement: S1 Fig — (A) Microglia were stimulated with Pam3CysSK4 (100 ng/ml) or LPS (100 ng/ml) for 24 h. Unstimulated cells served as a control. Microglia-conditioned supernatants were transferred to freshly isolated naïve γδ T cells. After 3 days, γδ T cells or microglia-conditioned supernatant only were supplemented with cortical TLR7KO neurons for additional 5 days. Neuronal cultures without γδ T cells in the presence of Pam3CysSK4 (100 ng/ml), LPS (100 ng/ml) or imiquimod (10 μg/ml) alone served as controls. Subsequently, cultures were immunostained with antibodies against CD3, NeuN, and neurofilament. NeuN-positive cells were quantified and expressed as relative neuronal viability. Each condition was performed in duplicate and averaged. Mean ± SEM of 3–5 individual experiments with ANOVA followed by Bonferroni multiple comparison post test, p<0.0001. (B) Primary cortical neurons derived from wild-type (WT) or TLR7KO mice were stimulated with loxoribine (1 mM). Untreated cells served as controls. After 5 days cultures were immunostained with antibodies against NeuN and neurofilament. NeuN-positive cells were quantified and expressed as relative neuronal viability. Each condition was performed in duplicate and averaged. Mean ± SEM of 2–3 individual experiments with ANOVA followed by Bonferroni multiple comparison post test, p<0.0001. (TIF) [file pone.0135898.s001.tif]
